# Estimating underreporting of leprosy in Brazil using a Bayesian approach

**DOI:** 10.1371/journal.pntd.0009700

**Published:** 2021-08-25

**Authors:** Guilherme L. de Oliveira, Juliane F. Oliveira, Júlia M. Pescarini, Roberto F. S. Andrade, Joilda S. Nery, Maria Y. Ichihara, Liam Smeeth, Elizabeth B. Brickley, Maurício L. Barreto, Gerson O. Penna, Maria L. F. Penna, Mauro N. Sanchez

**Affiliations:** 1 Departament of Computing, Federal Center of Technological Education of Minas Gerais, Belo Horizonte, Brazil; 2 Center of Data and Knowledge Integration for Health, Instituto Gonçalo Moniz, Fundação Oswaldo Cruz, Salvador, Brazil; 3 Department of Infectious Disease Epidemiology, London School of Hygiene & Tropical Medicine, London, United Kingdom; 4 Institute of Physics, Federal University of Bahia, Salvador, Brazil; 5 Institute of Collective Health, Federal University of Bahia, Salvador, Brazil; 6 Department of Non-communicable Disease Epidemiology, London School of Hygiene & Tropical Medicine, London, United Kingdom; 7 Health Data Research (HDR), London, United Kingdom; 8 Tropical Medicine Centre, University of Brasília, Brasília, Brazil; 9 Fiocruz School of Government, Brasília, Brazil; 10 Epidemiology and Biostatistics Department, Federal University Fluminense, Rio de Janeiro, Brazil; 11 Public Health Department, Federal University of Brasília, Brasília, Brazil; Adolfo Lutz Institute of Sao Jose do Rio Preto, BRAZIL

## Abstract

**Background:**

Leprosy remains concentrated among the poorest communities in low-and middle-income countries and it is one of the primary infectious causes of disability. Although there have been increasing advances in leprosy surveillance worldwide, leprosy underreporting is still common and can hinder decision-making regarding the distribution of financial and health resources and thereby limit the effectiveness of interventions. In this study, we estimated the proportion of unreported cases of leprosy in Brazilian microregions.

**Methodology/Principal findings:**

Using data collected between 2007 to 2015 from each of the 557 Brazilian microregions, we applied a Bayesian hierarchical model that used the presence of grade 2 leprosy-related physical disabilities as a direct indicator of delayed diagnosis and a proxy for the effectiveness of local leprosy surveillance program. We also analyzed some relevant factors that influence spatial variability in the observed mean incidence rate in the Brazilian microregions, highlighting the importance of socioeconomic factors and how they affect the levels of underreporting. We corrected leprosy incidence rates for each Brazilian microregion and estimated that, on average, 33,252 (9.6%) new leprosy cases went unreported in the country between 2007 to 2015, with this proportion varying from 8.4% to 14.1% across the Brazilian States.

**Conclusions/Significance:**

The magnitude and distribution of leprosy underreporting were adequately explained by a model using Grade 2 disability as a marker for the ability of the system to detect new missing cases. The percentage of missed cases was significant, and efforts are warranted to improve leprosy case detection. Our estimates in Brazilian microregions can be used to guide effective interventions, efficient resource allocation, and target actions to mitigate transmission.

## Author summary

Leprosy remains an important public health problem that affects vulnerable populations in low- and middle-income countries. In this study, we estimated how many cases were not detected in each Brazilian microregion by the surveillance system during the period of 2007 until 2015. Using a mathematical model, we found out that around 10% of the estimated number of cases occurring in the country in the study period were missed by the health system, and this represented over 33 thousand cases overall. These findings are important as undetected and therefore untreated cases can progress and lead to lifelong disabilities. In addition, missing cases prevents the health system from evaluating exposed contacts, which could lead to further delays in diagnosis. Describing the magnitude, location and correlates of leprosy underreporting at the microregional level will inform the efforts of policymakers and program managers in designing interventions and allocating resources to achieve leprosy control targets.

## Introduction

Leprosy is a Neglected Tropical Disease (NTD) caused by *Mycobacterium Leprae* that remains concentrated among individuals living under poor socioeconomic conditions in low- and middle-income countries [1, 2]. The WHO Global Leprosy Strategy 2016–2020 reinforces the need to strengthen leprosy surveillance and health information systems for programme monitoring and evaluation and to enhance early detection through active case finding in leprosy-endemic areas and among groups with increased risk (e.g., household contacts of leprosy patients [3]. Despite these recommendations, millions of individuals with leprosy remain undiagnosed and untreated worldwide [4].

Brazil is the second leading country in terms of the number of new leprosy cases detected, accounting for an estimated 14% of all new leprosy cases occurring worldwide [1]. Although the Brazilian notifiable disease registration system (SINAN) is considered largely reliable, there is evidence of leprosy underreporting in both endemic and non-endemic regions of the country [5–8]. Leprosy underreporting can occur for many reasons, including lack of knowledge or capacity of healthcare services or health professionals to diagnose and register new disease cases and lack of or resource limitations in regional and local leprosy control programmes.

Previous studies have estimated leprosy underreporting and corrected previous reporting rates for specific regions of Brazil. A 2001 study conducted in an endemic municipality in Northeast Brazil used a Bayesian spatial model to estimate leprosy underreporting among children and found a correlation between underreporting and multibacillary forms of the disease [9]. Another recent application of spatial-temporal Bayesian models was employed to estimate underreporting in the endemic state of Bahia in Northeast Brazil; the investigation correlated the presence of areas with underreporting (i.e., silent areas) with higher proportions of multibacillary cases and cases detected with Grade 2 leprosy-related physical disabilities (G2D) [10]. Despite those efforts, there is still no study providing national estimates of leprosy underreporting, which could enhance national surveillance systems and inform the targeting of leprosy detection and control policies in Brazil.

In this work, we used a Bayesian hierarchical model to estimate the micro-regional level predictive distribution for the true leprosy counts in Brazil for the period 2007–2015. The magnitude of leprosy underreporting was estimated based on the proportion of G2D as a direct indicator of delayed diagnosis and a proxy for the effectiveness of local leprosy surveillance program. The Bayesian framework used in this study allows for quantification of the uncertainty in correcting both the underreporting and the incidence rates.

## Materials and methods

### Ethics statement

This study was approved by the ethics committees of the University of Brasília (UnB) (1.822.125), Instituto Gonçalo Muniz—Fiocruz (1.612.302) and London School of Hygiene & Tropical Medicine (10580—1).

### Underreporting and incidence indicators

The detection of leprosy infection depends on the manifestation of symptoms in infected individuals. Further, individual immunological differences may influence the clinical presentation of symptoms, which only appear after a long incubation period estimated to range between two to seven years [11]. Individual cases can be diagnosed with no signs of leprosy-related physical disabilities (Grade 0 physical disabilities, G0D), with sensory loss in the absence of visible deformities or visual impairments (Grade 1 physical disabilities, G1D), or with visible deformities in the hands, feet, or eyes and/or severe visual impairment (G2D). Therefore, new cases of patients presenting high levels of disabilities is an indicator of delayed diagnosis and therefore reporting [12].

In addition to individual conditions, local endemicity, unfavourable socioeconomic factors (e.g., household crowding) and limited access to public health services influence the pattern of the disease incidence in the country. To account for their impacts on the spatial variability of leprosy incidence in each microregion, we included population-level socioeconomic indicators as variables in our analyses.

### Data source

We used data from the Brazilian National Notifiable Diseases Information System (SINAN). We collected the total and new yearly number of leprosy cases for each of the 558 microregions of Brazil from 2007 to 2015. We included only new cases of leprosy, which are defined, by the Ministry of Health, as cases with no previous treatment [13]. New cases of leprosy can be identified through active detection (e.g., contact testing with serological tests) or by passive detection when individuals spontaneously present to healthcare systems.

Additionally, to conduct our analysis, we utilized the following information: (1) the percentage of examined household contacts among the total registered household contacts in the microregion (*x*_1_), obtained from SINAN; (2) a proxy indicator of poverty defined as the percentage of the population at risk in the microregion that received benefits from the *Bolsa Família* conditional cash transfer programme (*x*_2_); (3) the percentage of the population at risk in the microregion registered in the Family Health Strategy (*x*_3_); (4) mean household density (i.e., number of people per household) in the microregion (*x*_4_), obtained from the Atlas of Human Development in Brazil (2010); and (5) urbanization, measured here by the percentage of people living in urban areas (*x*_5_), obtained from the Brazilian Institute of Geography and Statistics (IBGE 2010 demographic census). The percentage of new leprosy cases diagnosed with G2D (*w*), obtained from SINAN, was used as a proxy to estimate underreporting throughout the Brazilian microregions.

### Data curation

The analysis included leprosy cases detected between 2007 and 2015 with available spatial information related to the microregion of residence (*N* = 311, 970 new leprosy cases) and excluded cases from the *Fernando de Noronha* microregion (*N* = 2), as this island is located more than 500 km from the Brazilian mainland, which could introduce problems for the spatial analysis. Therefore, the spatial analysis was based on 311,968 cases from the 557 microregions of mainland Brazil.

To calculate the percentage of new leprosy cases diagnosed with G2D, we excluded from the analysis the observations with missing and “not evaluated” values from the variable that measures the grade of physical disabilities. They represented 10.35% of the total number of new cases registered. Therefore, 279,719 had information about disability grades and were considered in the further analysis.

The percentage of household contacts is given by the ratio of the number of examined contacts by the number of identified contacts in each microregion, multiplied by 100. We excluded the observations with missing values. As a result, we obtained a total of 267,160 individuals with valid information for our study.

### Statistical model for underreported leprosy data in Brazil

There are two main statistical approaches to deal with underreporting in disease surveillance and epidemiological studies. The first one refers to a censored Poisson likelihood function, allowing the estimation of the disease incidence rate and the probability of unreported cases in an area [14, 15]. The second approach relies on the specification of a hierarchical Poisson model, which assumes that all areas are potentially underreported, allowing the estimation of the disease incidence rate and the proportion of reported cases [16–24]. Therefore, the second approach, which we use in this study, allows a direct inference of the severity of underreporting instead of only inference on the chances of underreporting occurrence.

Both the censored and the hierarchical Poisson approaches rely on extra information (or “prior knowledge”) to supplement the partial information in the data. Therefore, the inference is usually made under the Bayesian framework, in which the extra information can be accommodated through appropriate prior distributions. Based on the prior knowledge we have available [25], we employed the hierarchical Poisson model under the Bayesian framework proposed by [23] in order to fit our model to the Brazilian leprosy data.

Under such a framework, in each microregion *i* (for *i* = 1, …, 557), the reported (observed) count *Y*_*i*_ is modelled as a Binomial random variable, where the number of trials is an unobserved Poisson variable *T*_*i*_ corresponding to the true number of cases that have been incompletely recorded. The true count generating process is modelled through the mean of the Poisson variable, denoted by *μ*_*i*_, and the reporting mechanism is modelled through the Binomial probability, denoted by *ϵ*_*i*_. Then, the basic structure for the hierarchical model is

$$\begin{array}{c} Y_{i}|T_{i},\epsilon_{i}\overset{ind}{∼}Binomial\left(T_{i},\epsilon_{i}\right);\;T_{i}|\mu_{i}\overset{ind}{∼}Poisson\left(\mu_{i}\right), \end{array}$$ (1)

for *i* = 1, …, 557. Since *T*_*i*_ is not observed, statistical inference is based on the marginal distribution of *Y*_*i*_ obtained from the joint model given in Eq (1), which is

$$\begin{array}{c} Y_{i}|\mu_{i},\epsilon_{i}\overset{ind}{∼}Poisson\left(\mu_{i}\epsilon_{i}\right). \end{array}$$ (2)

We assume that the mean expected new cases of leprosy, *μ*_*i*_, depends on five covariates *x*_1_ to *x*_5_ previously described as the proportion of household contacts examined, coverage of *Bolsa Família* Programme, coverage of the Family Health Strategy, the average number of people per household, and percentage of people living in urban areas; such that

$$\begin{array}{c} \text{log}\left(\mu_{i}\right)=\text{log}\left(P_{i}\right)+\beta_{0}+\sum_{k=1}^{5}\beta_{k}x_{ki}+\phi_{i}+\delta_{i},\;\text{for}\;\text{all}\;i=1,\ldots ,557. \end{array}$$ (3)

We also include the logarithm of the total population of the microregion, *log*(*P*_*i*_), as an offset, so that parameter *μ*_*i*_ can be interpreted as the leprosy incidence rate at area *i*. Additionally, in order to capture any residual variation in the leprosy incidence rate, we include a spatially structured random effect *ϕ*_*i*_ [26] and a local unstructured random effect *δ*_*i*_ in the log-linear predictor of *μ*_*i*_. The spatial structure is built based on the first-order neighbourhoods between the 557 microregions (i.e., two microregions are considered neighbours if they share a dividing edge).

To model *ϵ*_*i*_, the probability of reporting a new leprosy case at area *i*, we made use of the covariate *w* (the percentage of diagnosed new leprosy cases with G2D of physical disability) such that

$$\begin{array}{c} \text{logit}\left(\epsilon_{i}\right)=\alpha_{0}+\alpha_{1}w_{i}+\gamma_{i},\;\text{for}\;\text{all}\;i=1,\ldots ,557. \end{array}$$ (4)

As previously discussed, the covariate *w* acts as a *proxy* of the appropriate variable that accounts for notification efficiency of leprosy new cases in each microregion. An unstructured random effect *γ*_*i*_ is included in the logistic regression model of *ϵ*_*i*_ in order to account for potential effects of unobserved covariates that may influence the leprosy detection.

In the statistical literature, the joint model defined by Eqs (2), (3) and (4) is called the Poisson-Logistic (or Pogit) model. It is worth noting that the Pogit model provides a straightforward predictive analysis for the proportion of leprosy cases that were not observed (missed, i.e., not reported), denoted by *Z*_*i*_ = *T*_*i*_ − *Y*_*i*_. Given *μ*_*i*_ and *ϵ*_*i*_, *Z*_*i*_ can be predicted from the distribution $Z_{i}|\mu_{i},\epsilon_{i}\overset{ind}{∼}Poisson\left(\mu_{i}\left(1-\epsilon_{i}\right)\right)$ for all *i*.

Despite its appealing features, it is well-known in the literature that the Pogit model suffers from a lack of identifiability. This occurs because only the product *η*_*i*_ = *μ*_*i*_ *ϵ*_*i*_ is identified from the observed data since any other parameter combination, say $\tilde{\theta_{i}}$ and $\tilde{\epsilon_{i}}$, such that $\tilde{\theta_{i}}\tilde{\epsilon_{i}}=\eta_{i}$ yields the same likelihood function. In practice, such a concept means that additional information must be introduced in the model in order to distinguish between parameters *μ*_*i*_ and *ϵ*_*i*_. Such extra information can be provided by validation datasets, active search surveys or experts’ opinions. The source of information to be used will depend on which one is available for the specific practical situation one is dealing with.

To overcome the identifiability issue when fitting the Pogit model to the Brazilian leprosy count data, we follow the approach of [23] who analysed Brazilian tuberculosis data. Whenever the regression models for *μ*_*i*_ and *ϵ*_*i*_ do not share any common covariate, the lack of identifiability of the Pogit model relies on the confounding between the two intercepts *β*_0_ and *α*_0_. Under the Bayesian framework, the elicitation of an informative prior distribution for either *β*_0_ or *α*_0_ is an alternative to solve this issue.

As discussed in [23], by taking *w* and *x*_1_ to *x*_5_ as centred covariates, it provides that *β*_0_ and *α*_0_ are interpreted as the mean reported number of new leprosy cases (on the log scale) and the mean reporting rate (on the logistic scale) when the covariates are at their centring values, respectively. In this context, the appealing interpretation of *β*_0_ and *α*_0_ can be appropriately used to elicit an informative prior distribution for one of them, thus providing an identifiable Pogit model. For doing so, we rely on the information provided in [25] to define an informative prior distribution for the parameter *α*_0_. These authors performed an active search survey in some microregions of *Amazonas* State, North of Brazil, in 2012. We considered one of these microregions as the reference for centring the covariate *w*. Then, an informative Gaussian prior distribution was built for parameter *α*_0_ with basis on the reporting rate observed for such microregion in the study of [25]. A more detailed discussion is presented in the S1 and S2 Notes, along with the prior specification for the remaining parameters of our Bayesian model.

### Model implementation and validation

The model was implemented using the NIMBLE package [27] from R software [28]. For the Markov chain Monte Carlo (MCMC) scheme, two chains were used, each of them running a total of 600K iterations. The initial 200K iterations were discarded as burn-in period, and a lag of 200 iterations was considered to avoid correlated posterior samples. Trace plots of the MCMC samples were used to inspect convergence, and the potential scale reduction factor (PSRF), proposed by [29] was also computed. The PSRF was less than 1.10 for all regression coefficients and precision parameters, which is sufficient to indicate convergence to the target posterior distribution.

In addition to the inspection of the MCMC convergence, we assess the model validity by conducting a posterior predictive model checking as proposed in Chapter 6 of [30]. The idea of this approach is to look at the discrepancy between the observed data Y and the posterior predictive replicates of this data obtained from the fitted model. Results regarding the model validity are discussed in the S1 and S2 Notes, and they suggest that the model has no systematic issue (under or over-prediction) related to fitting the observed leprosy counts.

We used R software, geobr package, (MIT license https://ipeagit.github.io/geobr/) to create the maps produced in this work to visualise the spatial analysis, see [31, 32].

### Summary of the modelling results

To summarize, we applied the Bayesian model to correct the underreporting of leprosy cases. We estimate the posterior mean (Mean), the posterior standard deviation (SD) and the 90% highest posterior density intervals (90%-HPD) for all regression parameters defined on Eqs (3) and (4). When such intervals contain the value 0 it can be considered that the effect of the associated covariate is not significant. We also calculated the incidence rate ratio (IRR) for Poisson parameters and the odds ratio (OR) for logistic parameters with their respective 90%-HPD intervals. The IRR (or OR) indicates the effect in the mean incidence when a unity change occurs in the associated explanatory variable. The posterior summary measures for all model parameters are provided in the S1 Table and the main results are discussed in the following section.

## Results

### Distribution of incidence and level of detection of leprosy in the Brazilian landscape

From January 2007 to December 2015, a total of 312,114 new cases of leprosy were registered in SINAN, of which 7.6% had G2D at diagnosis. The highest mean incidences of leprosy in the period are concentrated in the North (42.95/100,000 inhabitants), Central-west (40.87/100,000 inhabitants) and Northeast (26.53/100,000 inhabitants) regions of Brazil. In the Central-West region, 46% were detected in Mato Grosso State. The states with the highest percentage of cases of G2D include Tocantins (12.33%), Maranhão (14.76%), Mato Grosso (12.06%), São Paulo (8.37%) and Rio Grande do Sul (6.21%). From 2007 to 2015, all Brazilian states showed a decreasing tendency in the number of leprosy cases, with the exception of Mato Grosso do Sul, who presented a 6% increase in leprosy incidence during this period.

**Fig 1:**
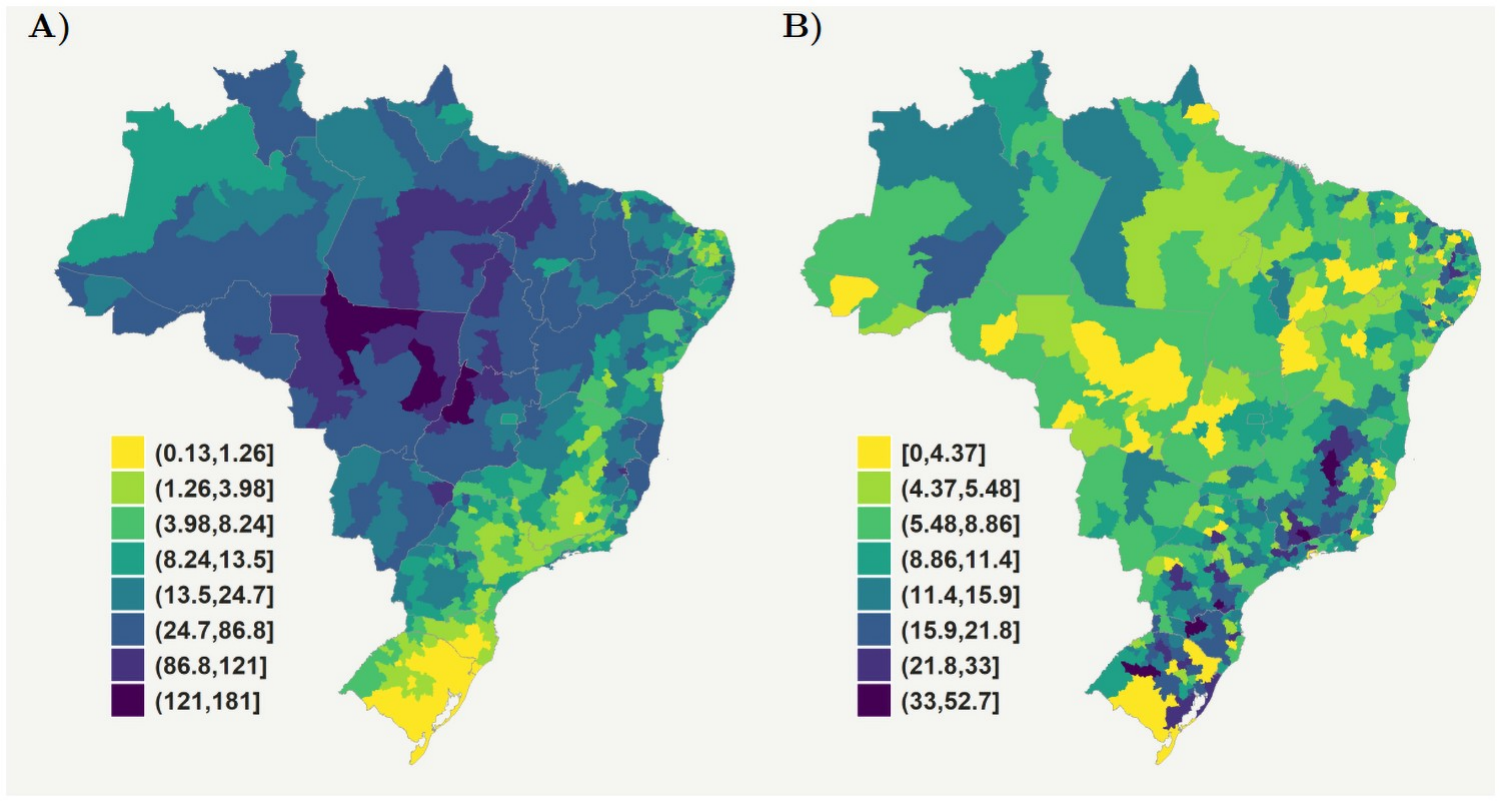
Brazilian microregions: (a) Observed leprosy incidence per 100,000 inhabitants in the period from 2007 to 2015; (b) Proportion of new leprosy cases diagnosed with Grade 2 of physical disabilities from 2007 to 2015. Similar plots at state level are presented in S1 Fig. Raw data are shown in this figure and available at https://github.com/cidacslab/Estimating-under-reporting-of-leprosy-in-Brazil.git [31]. We produced the maps using R software, geobr package [31, 32], (MIT license https://ipeagit.github.io/geobr/).

To proceed with a more granular analysis, we obtained, after data curation, a total of 267,160 cases with complete information for performing our analyses at the microregional level. The observed leprosy incidence per 100,000 in the period from 2007 to 2015 is higher in the microregions located in the Central-west and the Northern regions of the country (Fig 1), consistent with the full SINAN records. Different than in the full population, the proportion of new leprosy cases diagnosed with G2D in the sample with complete data are concentrated in the microregions located in the South and Southeast (Fig 1).

### The effect of social, poverty and living conditions on the spread of leprosy and underreporting levels

In an adjusted Poisson model accounting for the effects of covariates associated with leprosy incidence rate, we found that a unit increase in the proportion of household contacts examined was associated with a 0.75% increase in leprosy incidence (IRR = 1.0075, 90%HPD = 1.0027, 1.0122); a unit increase in the coverage of Bolsa Família Programme (i.e., as a proxy of poverty) was associated with a 1.41% increase in leprosy incidence (IRR = 1.0141, 90%HPD = 1.0039, 1.0252); and a unit increase in the percentage of people living in urban areas was associated with a 0.67% increase in leprosy incidence (IRR = 1.0067, 90%HPD = 1.0030, 1.0108) (see S1 Table and Fig 1). The mean household density was inversely associated with leprosy incidence (IRR = 0.6971, 90%HPD = 0.5414, 0.8680), and the coverage of the Family Health Strategy was not associated with leprosy rates (IRR = 1.0004, 90%HPD = 0.9972, 1.0032).

Based on the posterior result for the logistic model, we estimated that the percentage of new leprosy cases with G2D at diagnosis was associated with the reporting probability of leprosy (OR = 0.9417, 90%HPD = 0.9106, 0.9717), which means that a unit increase in this covariate reduces the probability of earlier reporting of a new case by 6% (See S1 Table).

**Fig 2:**
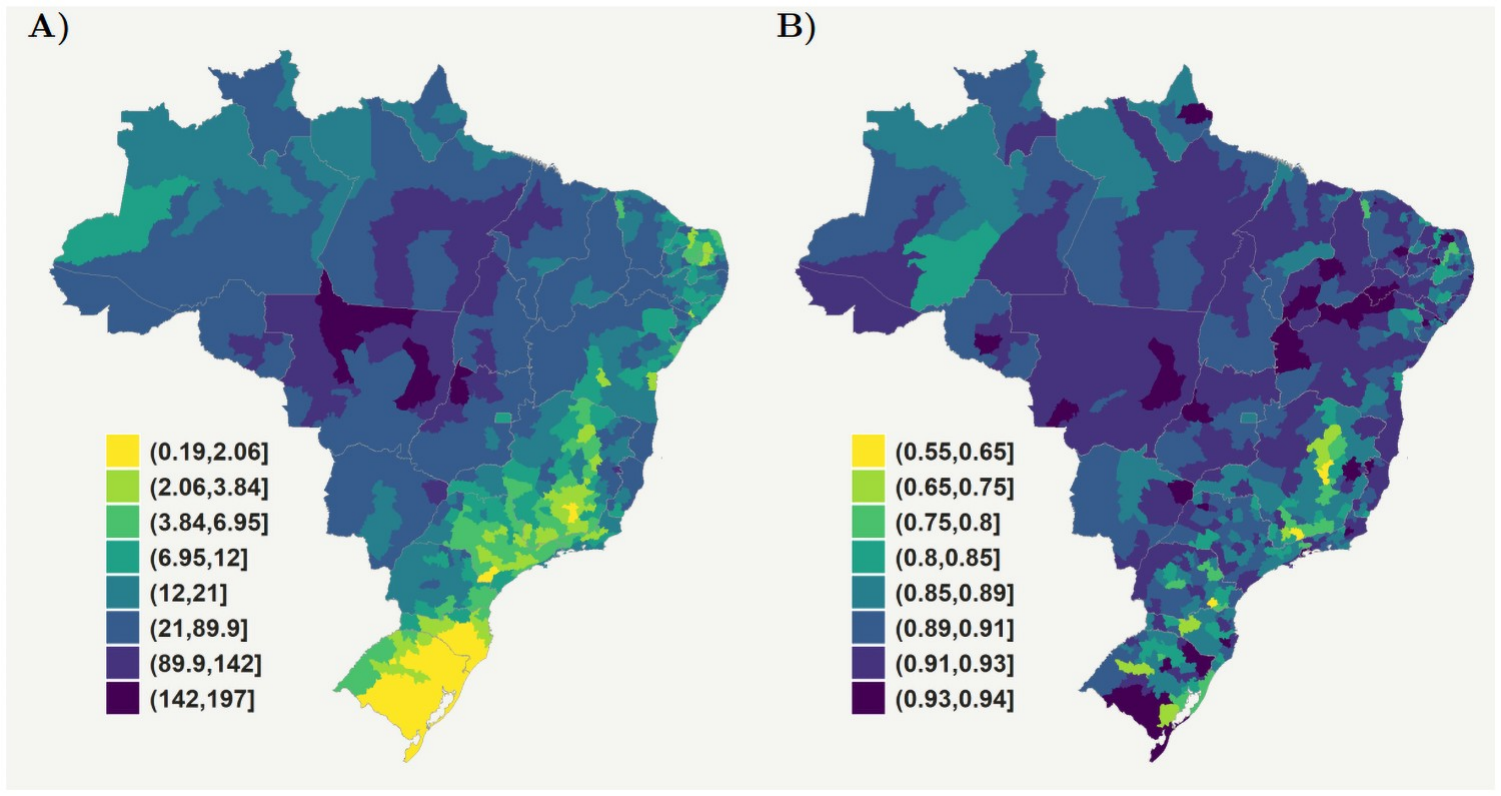
Brazilian microregions: (a) Mean leprosy incidence rate per 100,000 inhabitants in the period from 2007 to 2015 corrected by underreporting; (b) Estimated probability of reporting a leprosy case in each Brazilian microregion. Similar plots at state level are presented in the S1 Fig. Raw data are shown in this figure and available at https://github.com/cidacslab/Estimating-under-reporting-of-leprosy-in-Brazil.git [31]. We produced the maps using R software, geobr package [31, 32], (MIT license https://ipeagit.github.io/geobr/).

By using the percentage of G2D at the microregional level as a proxy for leprosy underreporting, we estimated that, on average, 33,252 (90%-HPD = (812;68,432)) or 9.6% (90%-HPD = (0.02%;19.8%)) of new cases of leprosy were not reported from 2007 to 2015 in Brazil (Table 1). The posterior estimates for the leprosy reporting probabilities show that Brazil’s South and Southwest regions present the lowest probability of reporting a case: 86.73% and 89.15% on average, respectively (Fig 2 and Table 1). We found that some microregions in the South and Southeast regions reported a less than 75% of the estimated number of new leprosy cases, which were notably concentrated in the State of Minas Gerais. In contrast, microregions in the states of Piauí and Pernambuco in the Northeast region and Mato Grosso in the Central-west region presented the highest estimates for reporting new leprosy cases, at 91.6% in each state (Table 1).

**Table 1:** Observed and estimated (posterior mean) number of new leprosy cases and overall leprosy detection rate for each Brazilian State for the period 2007–2015. The 90% highest posterior density (90%-HPD) interval is presented for the missed number o cases (*Z*) and for the detection rate.

Brazilian States	Observed number of cases (Y)	Estimated missed number of cases (Z)	Total corrected estimated number of cases (T)	Overall leprosy detection rate ($\frac{Y}{T}$%)
Rondônia	7,899	807 (19;1,662)	8,706	90.7 (82.6;99.8)
Acre	1,878	189 (3;385)	2,067	90.9 (83.0;99.8)
Amazonas	5,955	763 (11;1,588)	6,718	88.6 (78.9;99.8)
Roraima	1,235	150 (2;307)	1,385	89.2 (80.1;99.8)
Pará	34,041	3,337 (61;6,891)	37,378	91.1 (83.1;99;8)
Amapá	1,293	147 (4;310)	1,440	89.8 (80.7;99.7)
Tocantins	9,589	950 (15;980)	10,539	91.0 (83.0;99.8)
Maranhão	35,533	3,660 (144;7,502)	39,193	90.7 (82.6;99.6)
Piauí	11,446	1,050 (25;2,139)	12,496	91.6 (84.2;99.8)
Ceará	19,278	2,023 (64;4,181)	21,301	90.5 (82.2;99.7)
Rio Grande do Norte	2,532	299 (5;630)	2,831	89.5 (80.1;99.8)
Paraíba	6,093	647 (10;1,331)	6,740	90.4 (82.1;99.8)
Pernambuco	24,518	2,243 (44;4,567)	26,761	91.6 (84.3;99.8)
Alagoas	3,475	397 (8;809)	3,872	89.7 (81.1;99.8)
Sergipe	3,897	418 (10;859)	4,315	90.3 (81.9;99.7)
Bahia	23,968	2,327 (74;4,795)	26,295	91.2 (93.3;99.7)
Minas Gerais	14,117	2,005 (38;4,168)	16,122	87.6 (77.2;99.7)
Espírito Santo	8,291	786 (11;1,614)	9,077	91.3 (83.7;99.9)
Rio de Janeiro	15,117	1,787 (57;3,633)	16,904	89.4 (80.6;99.6)
São Paulo	15,835	2,091 (40;4,328)	17,926	88.3 (78.5;99.7)
Paraná	9,343	1,355 (24;2,799)	10,697	87.3 (76.9;99.7)
Santa Catarina	1,739	261 (4;536)	2,000	87.0 (76.4;99.8)
Rio Grande do Sul	1,331	218 (1;462)	1,549	85.9 (74.2;99.9)
Mato Grosso do Sul	6,486	772 (17;1,589)	7,258	89.4 (80.3;99.7)
Mato Grosso	24,902	2,285 (71;4,667)	27,187	91.6 (84.2;99.7)
Goiás	20,183	2,004 (44;4,131)	22,187	91.0 (83.0;99.8)
Distrito Federal	1,994	281 (6;584)	2,275	87.7 (77.3;99.7)
Total	311,968	33,252 (812;68,432)	345,220	90.4 (80.2;99.8)

## Discussion

This study used leprosy Grade 2 of physical disabilities and socioeconomic covariates to estimate underreporting of leprosy cases for each Brazilian microregion. We estimated that only 90.4% of new leprosy cases are reported in the country. Higher probabilities of underreporting are concentrated in microregions that diagnose leprosy less frequently, such as in the Southern Southeastern regions.

The number of missed cases is also a key finding of our study. Our estimates show that, on average, 33,252 new leprosy cases were missed by the surveillance system during the nine years of study. The mean reporting rate across the 557 microregions was 89.26% (SD = 0.0457), with some regions presenting critically low reporting, with the minimum value of 56.4%. On the other hand, some microregions indicated reporting of over 90%, with a maximum value of 93.94%. This heterogeneity also warrants further investigation from a policy, surveillance, and control program perspective and, from the methodological standpoint, regarding the model we have defined.

Although significant reductions in leprosy incidence have occurred in Brazil in recent decades, the proportion of cases with leprosy-associated physical disabilities have been increasing [33]. Our study found higher rates of underreporting and G2D at diagnosis in microregions with lower incidences of leprosy. Several factors can explain this result. First, it suggests underreporting occurs in microregions where there are later diagnoses and potentially fewer opportunities to start treatment. However, it may also reflect that the diagnosis of skin diseases may depend on the cultural importance given to skin lesions and health-seeking habits and/or recognition of leprosy symptoms among populations [34]. In addition, it may be partially explained by a lack of knowledge and training amongst healthcare workers who less frequently treat leprosy cases than those in regions with high endemicity [35].

The spatial variability in the observed mean incidence rate across Brazilian microregions over the study period can be partially attributed to the distribution of socioeconomic covariates affecting leprosy incidence. Our study shows that a higher incidence of leprosy is associated with the percentage of people living in urban areas and the percentage of household contacts experiencing poverty, measured by the coverage of the Bolsa Família Programme (BPF) [36], in an individual level study, showed that increased levels of deprivation and less schooling were associated with higher levels of new case detection. Considering that the BPF is a conditional cash transfer program targeting poor and extreme poor individuals, at municipal level, higher BPF coverage could act as a marker for higher levels of social and economic deprivation in the municipality [37]. In an individual-level study, [36] found that increased levels of coverage of the Bolsa Família Programme were associated with higher levels of new case detection. The proportion of examined contacts is a possible marker of the effectiveness of the local leprosy local control program in conducting contact examination. Furthermore, the importance of close contacts to high leprosy prevalence among the most impoverished Brazilians [38] suggests that underreporting of leprosy among contacts could lead to missed opportunities for early interventions and reduced transmission. The negative association between household crowding and leprosy incidence may be due to the high variability of household crowding across microregions and the presence of several microregions with an extremely high value for this variable but with a low incidence of leprosy.

Our study has limitations. To inform the priors for our parameters, we rely on data availability from areas that performed active surveillance to better report contacts and cases of leprosy. The reinforcement of such activities in other regions of Brazil with different social-economic conditions, particularly in more recent years, would improve the accuracy of our estimations. Still, with the model and proxy we used, we were able to capture a significant number of missed cases in the period, and this is concerning enough to make us recommend actions to improve surveillance efforts and health staff training.

Our findings reinforce the need to identify cases, ideally early in the course of disease progression, to break the transmission chain and prevent the development of physical disabilities. Further studies are needed to confirm the local drivers of underreporting. We recommend that local leprosy strategies pay special attention to areas where leprosy incidence correction for underreporting is most necessary. Reduction in the incidence of leprosy is the goal, as outlined in all plans leading to its control [3]. However, Brazil and other high-burden leprosy countries must consider estimating leprosy underreporting as part of the disease monitoring strategy, aiming to achieve national and international targets set forth by these plans.

## Supporting information

S1 Fig Observed leprosy incidence rate and the proportion of diagnoses of new leprosy cases diagnosed with Grade 2 of physical disabilities, together with their posterior means, for each Brazilian state, between 2007 to 2015. (PDF)

S2 Fig Exploratory analysis of the relation between the observed leprosy incidence and social-economic factors considered in our analysis. (PDF)

S1 Table Posterior summaries for the regression effects *β* and *α* and the model variance parameters for the Bayesian modelling applied to Brazilian leprosy data 2007–2015. (PDF)

S1 Note Model specification. (PDF)

S2 Note Model validation. (PDF)

## References

[1] World Health Organization. Global leprosy update, 2018: moving towards a leprosy-free world. Wkly Epidemiol Rec. 2019 Aug 30;94(35/36):389–411.

[2] Pescarini JM, Strina A, Nery JS, Skalinski LM, de Andrade KV, Penna ML, et al. Socioeconomic risk markers of leprosy in high-burden countries: A systematic review and meta-analysis. PLoS neglected tropical diseases. 2018 Jul 9;12(7):e0006622. doi: 10.1371/journal.pntd.0006622 29985930 PMC6053250

[3] World Health Organization. Global Leprosy Strategy 2016-2020: accelerating towards a leprosy-free world-Operational manual.

[4] Smith WC, van Brakel W, Gillis T, Saunderson P, Richardus JH. The missing millions: a threat to the elimination of leprosy. PLoS Negl Trop Dis. 2015 Apr 23;9(4):e0003658. doi: 10.1371/journal.pntd.0003658 25905706 PMC4408099

[5] Galvao PR, Ferreira AT, Maciel MD, De Almeida RP, Hinders D, Schreuder PA, et al. An evaluation of the Sinan health information system as used by the Hansen’s disease control programme, Pernambuco State, Brazil. Leprosy review. 2008 Jun 1;79(2):171–83. 18711939

[6] Façanha MC, Pinheiro AC, Lima JR, Ferreira ML, Teixeira GF, Rouquayrol MZ. Hanseníase: subnotificação de casos em Fortaleza-Ceará, Brasil. Anais brasileiros de dermatologia. 2006 Aug;81(4):329–33. doi: 10.1590/S0365-05962006000400004

[7] Bernardes Filho F, Paula NA, Leite MN, Abi-Rached TL, Vernal S, Silva MB, et al. Evidence of hidden leprosy in a supposedly low endemic area of Brazil. Memórias do Instituto Oswaldo Cruz. 2017 Dec;112(12):822–8. doi: 10.1590/0074-02760170173 29211243 PMC5719551

[8] Barreto JG, Guimarães LD, Frade MA, Rosa PS, Salgado CG. High rates of undiagnosed leprosy and subclinical infection amongst school children in the Amazon Region. Memórias do Instituto Oswaldo Cruz. 2012 Dec;107:60–7. doi: 10.1590/S0074-02762012000900011 23283455

[9] Souza WV, Barcellos CC, Brito AM, Carvalho MS, Cruz OG, Albuquerque MF, et al. Empirical bayesian model applied to the spatial analysis of leprosy occurrence. Revista de saude publica. 2001 Oct 1;35(5):474–80. doi: 10.1590/s0034-89102001000500011 11723520

[10] Souza CD, Santos FG, Marques CD, Leal TC, Paiva JP, Araújo EM. Spatial study of leprosy in Bahia, Brazil, 2001-2012: an approach based on the local empirical Bayesian model. Epidemiologia e Serviços de Saúde. 2018 Nov 29;27:e2017479. 30517350 10.5123/S1679-49742018000400013

[11] Moreira MB, Costa Neto MM. Controle da hanseníase na atenção básica: guia prático para profissionais da equipe da saúde da família. Brasília: Ministério da Saúde. 2001. Available at: https://www.yumpu.com/pt/document/view/14734195/controle-da-hanseniase-na-atencao-basica-guia-pratico.

[12] Nobre ML, Illarramendi X, Dupnik KM, Hacker MD, Nery JA, Jerônimo SM, et al. Multibacillary leprosy by population groups in Brazil: Lessons from an observational study. PLoS neglected tropical diseases. 2017 Feb 13;11(2):e0005364. doi: 10.1371/journal.pntd.0005364 28192426 PMC5325588

[13] Brasil. Diretrizes para vigilância, atenção e eliminação da Hanseníase como problema de saúde pública: manual técnico-operacional. Available at: http://portalsaude.saude.gov.br/images/pdf/2016/fevereiro/04/diretrizes-eliminacao-hanseniase-4fev16-web.pdf.

[14] Bailey TC, Carvalho MS, Lapa TM, Souza WV, Brewer MJ. Modeling of under-detection of cases in disease surveillance. Annals of Epidemiology. 2005 May 1;15(5):335–43. doi: 10.1016/j.annepidem.2004.09.013 15840546

[15] de Oliveira GL, Loschi RH, Assunção RM. A random-censoring Poisson model for underreported data. Statistics in medicine. 2017 Dec 30;36(30):4873–92. doi: 10.1002/sim.7456 29067731

[16] Moreno E, Giron J. Estimating with incomplete count data A Bayesian approach. Journal of Statistical Planning and Inference. 1998 Jan 5;66(1):147–59. doi: 10.1016/S0378-3758(97)00073-6

[17] Whittemore AS, Gong G. Poisson regression with misclassified counts: application to cervical cancer mortality rates. Journal of the Royal Statistical Society: Series C (Applied Statistics). 1991 Mar;40(1):81–93. 12157994

[18] Stamey JD, Young DM, Boese D. A Bayesian hierarchical model for Poisson rate and reporting-probability inference using double sampling. Australian & New Zealand Journal of Statistics. 2006 Jun;48(2):201–12. doi: 10.1111/j.1467-842X.2006.00434.x

[19] Papadopoulos G, Silva JS. Identification issues in some double-index models for non-negative data. Economics Letters. 2012 Oct 1;117(1):365–7. doi: 10.1016/j.econlet.2012.06.001

[20] Dvorzak M. and Wagner H. (2016). Dvorzak M, Wagner H. Sparse Bayesian modelling of underreported count data. Statistical Modelling. 2016 Feb;16(1):24–46. doi: 10.1177/1471082X15588398

[21] Shaweno D, Trauer JM, Denholm JT, McBryde ES. A novel Bayesian geospatial method for estimating tuberculosis incidence reveals many missed TB cases in Ethiopia. BMC infectious diseases. 2017 Dec;17(1):1–8. doi: 10.1186/s12879-017-2759-0 28969585 PMC5625624

[22] Schmertmann CP, Gonzaga MR. Bayesian estimation of age-specific mortality and life expectancy for small areas with defective vital records. Demography. 2018 Aug;55(4):1363–88. doi: 10.1007/s13524-018-0695-2 29978339

[23] Stoner O, Economou T, da Silva GD. A hierarchical framework for correcting under-reporting in count data. Journal of the American Statistical Association. 2019 Apr 30. doi: 10.1080/01621459.2019.1573732

[24] de Oliveira GL, Argiento R, Loschi RH, Assunçao RM, Ruggeri F, Branco MD. Bias Correction in Clustered Underreported Data. Bayesian Analysis. 2021.

[25] Cunha C, Pedrosa VL, Dias LC, Braga A, Chrusciak-Talhari A, Santos M, et al. A historical overview of leprosy epidemiology and control activities in Amazonas, Brazil. Revista da Sociedade Brasileira de Medicina Tropical. 2015 Jun;48:55–62. doi: 10.1590/0037-8682-0103-2013 26061371

[26] Besag J, York J, Mollié A. Bayesian image restoration, with two applications in spatial statistics. Annals of the institute of statistical mathematics. 1991 Mar;43(1):1–20. doi: 10.1007/BF00116466

[27] de Valpine P, Turek D, Paciorek CJ, Anderson-Bergman C, Lang DT, Bodik R. Programming with models: writing statistical algorithms for general model structures with NIMBLE. Journal of Computational and Graphical Statistics. 2017 Apr 3;26(2):403–13. doi: 10.1080/10618600.2016.1172487

[28] Team RC. R: A language and environment for statistical computing. R Foundation for Statistical Computing, Vienna, Austria. ISBN 3-900051-07-0, 2015. URL:https://www.R-project.org/

[29] Brooks SP, Gelman A. General methods for monitoring convergence of iterative simulations. Journal of computational and graphical statistics. 1998 Dec 1;7(4):434–55. doi: 10.1080/10618600.1998.10474787

[30] Gelman A, Carlin J, Stern H, Dunson D, Vehtari A, Rubin D. Bayesian Data Analysis (Chapman and Hall/CRC Texts in Statistical Science) (Third ed.), London: Chapman and Hall/CRC. 2014.

[31] Pereira RH, Gonçalves CN. geobr: Loads Shapefiles of Official Spatial Data Sets of Brazil. GitHub repository. 2019.

[32] Oliveira G.L., Oliveira J.F., Pescarini J.S., Andrade R.F., Nery J.M., Ichihara M.Y., et al. Estimating Under Reporting of Leprosy in Brazil using a Bayesian Approach. *GitHub repository: Estimating Under Reporting of Leprosy in Brazil using a Bayesian Approach*, 2021. 10.1371/journal.pntd.0009700 PMC8423270 34432805

[33] Pescarini JM.; Teixeira CSS., Silva NB., Sanchez MN., Natividade MS., Rodrigues LC. et al. Epidemiological characteristics and temporal trends of new leprosy cases in Brazil: 2006 to 2017. Cadernos de Saúde Pública. 2021. doi: 10.1590/0102-311x00130020 34346981

[34] Penna ML, Reydeoliveira MV, Penna GO. The epidemiological behaviour of leprosy in Brazil. Leprosy review. 2009 Sep 1;80(3):332–45. doi: 10.47276/lr.80.3.332 19961107

[35] Souza CD, Tavares DL, Tavares CM, Almeida AG, Accioly SM, Paiva JP, et al. Physical disabilities due to leprosy in Alagoas State, Northeast Brazil: a temporal and spatial modeling. Revista da Sociedade Brasileira de Medicina Tropical. 2019;52. doi: 10.1590/0037-8682-0540-2018 31340360

[36] Nery JS, Ramond A, Pescarini JM, Alves A, Strina A, Ichihara MY, et al. Socioeconomic determinants of leprosy new case detection in the 100 Million Brazilian Cohort: a population-based linkage study. The Lancet Global Health. 2019 Sep 1;7(9):e1226–36. doi: 10.1016/S2214-109X(19)30260-8 31331811 PMC6688099

[37] de Souza PH, Osorio RG, Paiva LH, Soares S. Os efeitos do Programa Bolsa Família sobre a pobreza e a desigualdade: Um balanço dos primeiros quinze anos. Texto para discussão; 2019.

[38] Teixeira CS, Pescarini JM, Alves FJ, Nery JS, Sanchez MN, Teles C, et al. Incidence of and factors associated with leprosy among household contacts of patients with leprosy in Brazil. JAMA dermatology. 2020 Jun 1;156(6):640–8. doi: 10.1001/jamadermatol.2020.0653 32293649 PMC7160739

