# Supplementary material for: Estimating underreporting of leprosy in Brazil using a Bayesian approach
Source: PLoS Negl Trop Dis. 2021 Aug 25;15(8):e0009700. doi: 10.1371/journal.pntd.0009700 (PMC8423270; doi:10.1371/journal.pntd.0009700)
Supplement: S1 Fig — (PDF) [file pntd.0009700.s001.pdf]

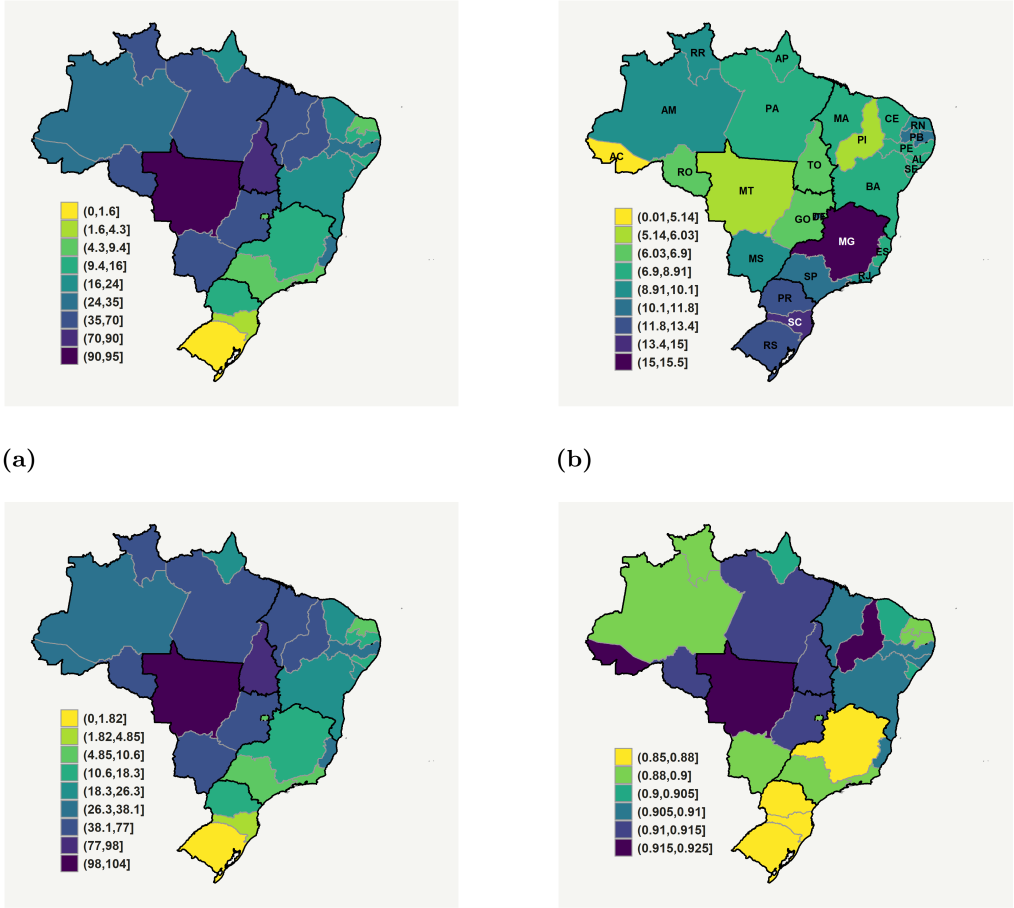

(a) **(b)**

(c) **(d)**

Supplementary Figure 1. Brazilian States: (a) Observed leprosy incidence rate per 100,000 inhabitants between 2007 to 2015; (b) Proportion of diagnoses of new leprosy cases diagnosed with Grade 2 of physical disabilities between 2007 to 2015; (c) Posterior mean for the leprosy incidence rate per 100,000 inhabitants between 2007 to 2015 corrected by underreporting; (d) Posterior mean for the probability of reporting a leprosy case in each Brazilian State. The black line highlights the five Brazilian macroregions (North, Northeast, Central-west, Southeast and South). In the map the abbreviations stands for: Rondônia (RO), Acre (AC), Amazonas (AM), Roraima (RR), Pará (PA), Amapá (AP), Tocantins (TO), Maranhão (MA), Piauí (PI), Ceará (CE), Rio Grande do Norte (RN), Paraíba (PB), Pernambuco (PE), Alagoas (AL), Sergipe (SE), Bahia (BA) Minas Gerais (MG), Espírito Santo (ES), Rio de Janeiro (RJ) São Paulo (SP), Paraná (PR), Santa Catarina (SC), Rio Grande do Sul (RS), Mato Grosso do Sul (MS), Mato Grosso (MT), Goiás (GO), Distrito Federal (DF). We produced the maps using R software, geobr package [31] (MIT license https://ipeagit.github.io/geobr/).
