# Supplementary material for: Estimating underreporting of leprosy in Brazil using a Bayesian approach
Source: PLoS Negl Trop Dis. 2021 Aug 25;15(8):e0009700. doi: 10.1371/journal.pntd.0009700 (PMC8423270; doi:10.1371/journal.pntd.0009700)
Supplement: S2 Fig — (PDF) [file pntd.0009700.s002.pdf]

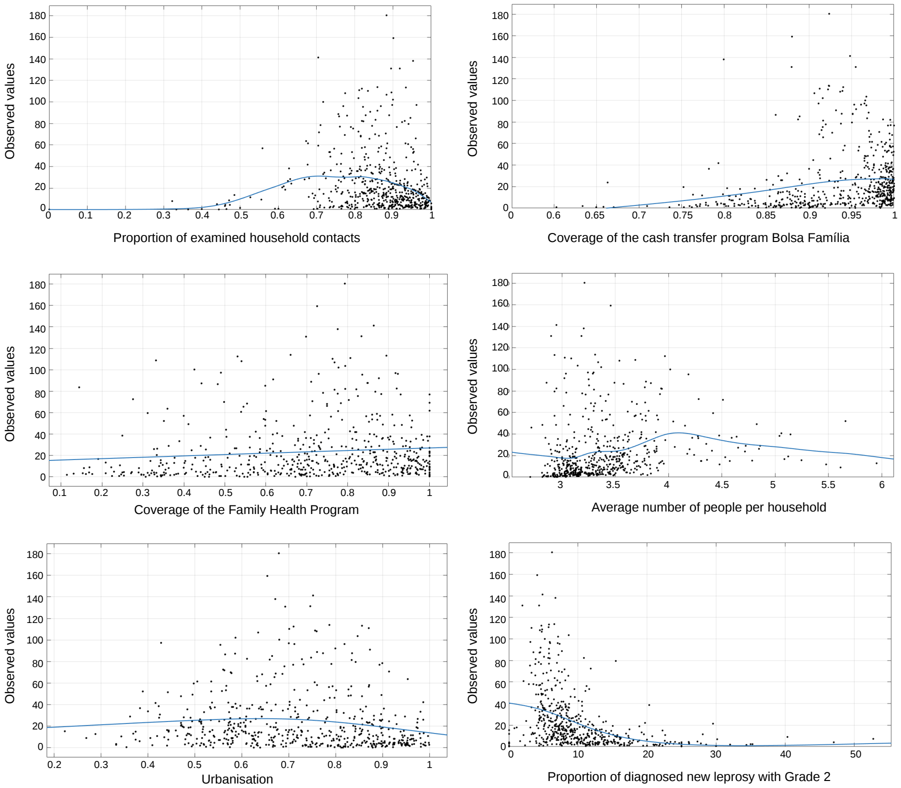


**Supplementary Figure 2. Exploratory analysis of the relation between the observed leprosy incidence and social-economic factors considered in our analysis.** Relations between the observed values of incidence of leprosy and the variables considered in the model (raw data represented by black dots) with spline smoothing (blue lines).
