# Supplementary material for: Estimating underreporting of leprosy in Brazil using a Bayesian approach
Source: PLoS Negl Trop Dis. 2021 Aug 25;15(8):e0009700. doi: 10.1371/journal.pntd.0009700 (PMC8423270; doi:10.1371/journal.pntd.0009700)
Supplement: S1 Table — (PDF) [file pntd.0009700.s003.pdf]

Supplementary Table 1. Posterior summaries for the regression effects $\boldsymbol{\beta}$ and $\boldsymbol{\alpha}$ and the model variance parameters; Brazilian leprosy data 2007-2015. For each parameter we provide the posterior mean (Mean), the posterior standard deviation (SD) and the 90% highest posterior density (90%-HPD) interval. The incidence rate ratio (IRR) for Poisson parameters and the odds ratio (OR) for logistic parameters are also provided, along with the associated 90%-HPD interval.

Poisson	Covariate Name	Mean	SD	95%-HPD	IRR (95%-HPD)
β_0	mean reported number of leprosy cases (log scale)	-8.8992	0.0416	(-8.9223, -8.8140)	-
β_1	percentage of household contacts examinated	0.0075	0.0029	(0.0019, 0.0131)	1.0075 (1.0019, 1.0132)
β_2	coverage of the Bolsa Família Programme	0.0140	0.0064	(0.0016, 0.0259)	1.0141 (1.0016, 1.0262)
β_3	coverage of the Family Health Strategy	0.0004	0.0018	(-0.0032, 0.0040)	1.0004 (0.9968, 1.0040)
β_4	average number of people per household	-0.3608	0.1403	(-0.6189, -0.0777)	0.6971 (0.5261, 0.9077)
β_5	percentage of people living in urban areas	0.0067	0.0023	(0.0023, 0.0114)	1.0067 (1.0023, 1.0114)
ν	spatial effect precision parameter	0.9783	0.0942	(0.7992, 1.1630)	-
σ_δ	unstructured effect variance parameter	0.0842	0.0157	(0.0555, 0.1149)	-
**Logistic**	**Covariate Name**	**Mean**	**SD**	**95%-HPD**	**OR (95%-HPD)**
α_0	mean reporting rate (logistic scale)	2.4452	0.3757	(1.6828,3.2062)	-
α_1	percentage of diagnosed new leprosy cases with G2D	-0.0601	0.0204	(-0.1002,-0.0199)	0.9417 (0.9047, 0.9803)
σ_γ	unstructured effect variance parameter	0.5921	0.3067	(0.1192, 1.1789)	-
