# Supplementary material for: Estimating underreporting of leprosy in Brazil using a Bayesian approach
Source: PLoS Negl Trop Dis. 2021 Aug 25;15(8):e0009700. doi: 10.1371/journal.pntd.0009700 (PMC8423270; doi:10.1371/journal.pntd.0009700)
Supplement: S1 Note — (PDF) [file pntd.0009700.s004.pdf]

Supplementary Note 1

Model Specification

In this section we present in details the prior specification for all model parameters. As mentioned in the manuscript, to overcome the identifiability issue when fitting the Pogit model to the Brazilian leprosy count data, we follow the approach used by [1] to analyze Brazilian tuberculosis data. Whenever the regression models of μ_i and ϵ_i do not share any common covariate, the identifiability issue relies on the confounding between the two intercepts α_0 and β_0. Then, special attention must be given to their prior distributions as detailed in the following.

As discussed in [1], by taking x_1 to x_5 and w as centered covariates it provides that β_0 and α_0 are, respectively, interpreted as the mean reported number of leprosy new cases (on the log scale) and the mean reporting rate (on the logistic scale) when the covariates are at their centering values. In this context, the appealing interpretation of α_0 and β_0 can be appropriately used to elicit an informative prior distribution for one of them, thus providing an identifiable Pogit model.

We found that there is no study seeking to estimate the detection rate of leprosy in a national level between the period of 2007 to 2015. If such a study was available, we could center the covariate w with respect to its observed mean and then elicit an appropriate informative prior for parameter α_0, which, in this case, could be interpreted as the overall mean reporting rate. In the work by [3], the authors present results of an active search survey, performed in 2012, in some municipalities of the state of *Amazonas*, Northern of Brazil. Based on their findings, the microregion *Rio Preto da Eva* (RPE), composed by the municipalities *Rio Preto da Eva* and *Presidente Figueiredo*, showed reporting rates of approximately 91% when comparing the total cases registered by the local health centers. In particular, the microregion of RPE is located in the metropolitan region of *Manaus*, capital of the State of *Amazonas*, and it is one of the most developed regions in northwest of Brazil.

As far as we know, this is the most trustful available study about the level of leprosy underreporting in Brazil. Therefore, we rely on the information provided for this microregion to define an informative prior distribution for parameter α_0. To do so, we center the covariate w with respect to its observed value for *Rio Preto da Eva* so that the parameter α_0, on the logistic scale, represents the average level of notification of leprosy cases in such microregion. Moreover, we assume that the information for the level of notification for the period from 2007 to 2015 can be represented by findings of [3] for year 2012. We then specified a $N(2.5, 0.3)$ as the prior distribution for the parameter, which provides an *a priori* average level of reporting of approximately 91% for this microregion, on the logistic scale. The notation $N(\mu, \sigma)$ represents a Gaussian (Normal) distribution with mean μ and standard deviation σ. The choice for the standard deviation was guided by the sensitivity studies presented by [1]. They suggest that the model is robust in terms of quantifying uncertainty as long as the practitioner specifies a prior distribution for α_0 that is informative without the need of being a degenerated one (a prior with null variance). Through an *a priori* predictive analysis in our application, the variation expressed by the prior distribution $N(2.5, 0.3)$ provides values for ϵ in the microregion RPE with high concentration (about 90%) between 88.2% and 95.1%, reflecting well our belief regarding the average level of reporting used as a reference for the period.

We now provide the prior distribution for parameter β_0. As an informative prior was already elicited for the other model intercept, α_0, there is no need for doing the same in relation to β_0. Nevertheless, to avoid the generation of unrealistic values for the leprosy incidence rate (especially quite elevated values), we also follow the approach proposed in [1]. Each covariate x_1 to x_5 was centered with relation to its observed mean such that β_0 is interpreted as the mean reported number of leprosy cases, on the logarithmic scale.

Then, we assume a prior $N(-8, 1)$ for β_0 to represent our belief that is not plausible a very high value (such as over 1 million) for the total number of new leprosy cases. For each of the remaining regression coeficients, α_1 and $\beta_1, \ldots, \beta_5$, we elicit a Normal prior distribution $N(0, 10)$.

The model specification is completed with the following prior distributions for the random effects and their precision terms. As it is usual in the literature, the prior distribution of ϕ_i, with precision parameter ν, is represented by an intrinsic conditional autoregressive (iCAR) model [4]. Here, a neighbor of an area i was defined as any $i' \neq i$ sharing a geographical boundary with i. The unstructured effect γ_i and the local effect δ_i are assumed to have a Normal prior distribution $N(0, \sigma_\gamma)$ and $N(0, \sigma_\delta)$, respectively. For each of the precision parameters ν, $1/\sigma_\delta^2$ and $1/\sigma_\gamma^2$ we elicited a Gamma distribution $G(1, 1)$.

References

1. Stoner, O; Economou, T; Drummond, G. (2019). A Hierarchical Framework for Correcting Under-Reporting in Count Data. *Journal of the American Statistical Association* , DOI 10.1080/01621459.2019.1573732.

2. Oliveira, G.L.; Argiento, R.; Loschi, R.H.; Assunção, R.M.; Ruggeri, F. and Branco, M.D. (2020) Bias Correction in Clustered Underreported Data. Bayesian Analisys, advance publication, 25 September 2020. doi:10.1214/20-BA1244.

3. Cunha, C., Pedrosa, V.L., Dias, L.C., Braga, A., Chrusciak-Talhari, A., Santos, M., Penna, G.O., Talhari, S. and Talhari, C. (2015). A historical overview of leprosy epidemiology and control activities in Amazonas, Brazil. *Revista da Sociedade Brasileira de Medicina Tropical*, **48**(Suppl I):55–62.

4. Besag, J., York, J., and Mollié, A. (1991). Bayesian image restoration, with two applications in spatial statistics. *Annals of the Institute of Statistical Mathematics*, **43**(1), 1–20.

5. Gelman A, Carlin J, Stern H, Dunson D, Vehtari A, Rubin D. Bayesian Data Analysis (Chapman and Hall/CRC Texts in Statistical Science) (Third ed.), London: Chapman and Hall/CRC. 2014.
