# Supplementary material for: Estimating underreporting of leprosy in Brazil using a Bayesian approach
Source: PLoS Negl Trop Dis. 2021 Aug 25;15(8):e0009700. doi: 10.1371/journal.pntd.0009700 (PMC8423270; doi:10.1371/journal.pntd.0009700)
Supplement: S2 Note — (PDF) [file pntd.0009700.s005.pdf]

Supplementary Note 2

Model Validation

Besides inspecting convergence of the MCMC (Markov chain Monte Carlo) samples, we assess the model validity by conducting a posterior predictive model checking (see Chapter 6 of [1]) where the goal is to look at the discrepancy between the observed data Y and the posterior predictive replicates $\tilde{Y}$ of this data obtained from the fitted model. Consider that the posterior predictive distribution for a replicate $\tilde{Y}$ related to the observed leprosy count Y is the distribution $p(\tilde{Y}|Y)$. The point is then whether the actual observation Y can be considered an extreme value with respect to $p(\tilde{Y}|Y)$ and if so, this indicates poor model performance.

Supplementary Figure A 1(a) shows scatterplots comparing the mean predicted values for Y (the observed underreported leprosy counts) with respect to their corresponding true values, showing that the fitted model is adequate to detect the observed values very well. Supplementary Figure A 1(b) shows a scatterplot of the difference between the lower (blue) and upper (green) limits of the 95% posterior predictive intervals of the replicates $\tilde{Y}$ and the corresponding observed values Y. The predictive intervals are symmetrically centered on the observed values, suggesting that the model has no systematic issue (under or over-prediction) related to fitting the observed leprosy counts and implying good prediction accuracy.

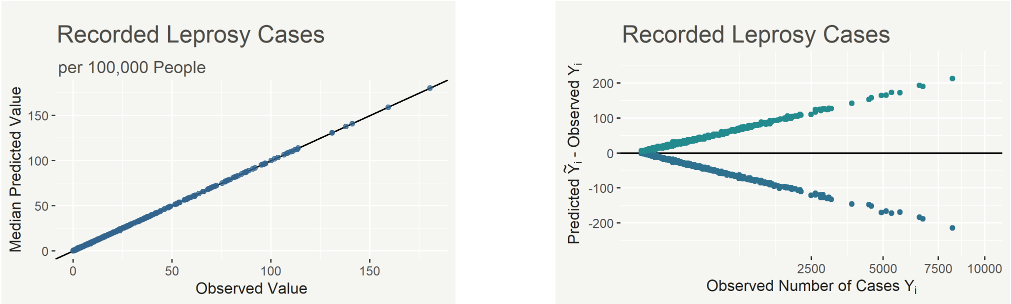

(a) **(b)**

Supplementary Figure A 1. Scatterplot comparing the observed counts to the median predicted counts from the model (a) and scatterplot of differences between the lower (blue) and upper (green) limits of the 90% predictive interval related to each observed count (b).

Supplementary References

1. Gelman A, Carlin J, Stern H, Dunson D, Vehtari A, Rubin D. Bayesian Data Analysis (Chapman and Hall/CRC Texts in Statistical Science) (Third ed.), London: Chapman and Hall/CRC. 2014.
